# Retinal–Carotenoid Interactions in a Sodium-Ion-Pumping Rhodopsin: Implications on Oligomerization and Thermal Stability

**DOI:** 10.1021/acs.jpcb.2c07502

**Published:** 2023-03-01

**Authors:** Mihir Ghosh, Ramprasad Misra, Sudeshna Bhattacharya, Koushik Majhi, Kwang-Hwan Jung, Mordechai Sheves

**Affiliations:** † Department of Molecular Chemistry and Materials Science, Weizmann Institute of Science, Rehovot 76100, Israel; ‡ Department of Life Science and Institute of Biological Interfaces, Sogang University, Seoul 04107, South Korea

## Abstract

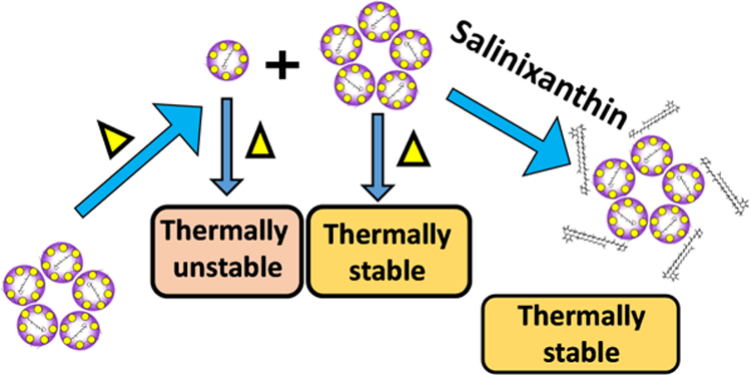

Microbial rhodopsin (also called retinal protein)–carotenoid conjugates represent a unique class of light-harvesting (LH) complexes, but their specific interactions and LH properties are not completely elucidated as only few rhodopsins are known to bind carotenoids. Here, we report a natural sodium-ion (Na^+^)-pumping *Nonlabens (Donghaeana) dokdonensis* rhodopsin (DDR2) binding with a carotenoid salinixanthin (Sal) to form a thermally stable rhodopsin–carotenoid complex. Different spectroscopic studies were employed to monitor the retinal–carotenoid interaction as well as the thermal stability of the protein, while size-exclusion chromatography (SEC) and homology modeling are performed to understand the protein oligomerization process. In analogy with that of another Na^+^-pumping protein *Krokinobacter eikastus* rhodopsin 2 (KR2), we propose that DDR2 (studied concentration range: 2 × 10^–6^ to 4 × 10^–5^ M) remains mainly as a pentamer at room temperature and neutral pH, while heating above 55 °C partially converted it into a thermally less stable oligomeric form of the protein. This process is affected by both the pH and concentration. At high concentrations (4 × 10^–5^ to 2 × 10^–4^ M), the protein adopts a pentamer form reflected in the excitonic circular dichroism (CD) spectrum. In the presence of Sal, the thermal stability of DDR2 is increased significantly, and the pigment is stable even at 85 °C. The results presented could have implications in designing stable rhodopsin–carotenoid antenna complexes.

## Introduction

Microbial retinal proteins contain an all-*trans* retinal chromophore covalently bound to a lysine residue through a protonated Schiff base (PSB) formation, which is used to harvest light.^1−13^ While animal rhodopsins (type-II) are classified as G-protein coupled receptors (GPCRs), microbial rhodopsins have a wide range of functions, including light gated proton (H^+^), chloride (Cl^–^), and sodium (Na+) ion pumps, ion channels, and phototaxis sensors.^1−4^ There are two light-harvesting systems generally known to exist in nature.^14−16^ The common one is chlorophyll-based light harvesters, and the second one is retinal-based. There are several other photoreceptor proteins known in nature that use flavins and open-tetrapyrroles as chromophores.^17,18^ Recently, it was revealed that a retinal-based light-harvesting protein, named xanthorhodopsin (acronymed xR), contains a salinixanthin (Sal) carotenoid in addition to all-*trans* retinal.^14^ Although the chlorophyll-based LH complexes have widely been studied,^15,16^ studies of retinal–carotenoid light-harvesting systems are scarce mainly because only few rhodopsins were reported to bind carotenoids. Only proton-pumping microbial rhodopsins, such as xR, thermophilic rhodopsin (TR), and *Gloebacter* rhodopsin (GR), were reported to bind carotenoid.^14,19−24^ The presence of the glycine (Gly) residue in the vicinity of the retinal ring was reported to be common in rhodopsins that bind Sal.^24−26^

Recently, a light-driven sodium ion pump was discovered in the marine flavobacterium *Krokinobacter eikastus*, which possesses two rhodopsins (KR1 and KR2).^23^ While KR1 is a typical proton pump, KR2 pumps sodium ions outward. There is evidence that the sodium-ion-pumping rhodopsin can also pump lithium ions, but it becomes a proton pump in potassium chloride or salts of larger cations. As per later studies,^25^ replacement of the Thr216 residue with the Gly of the sodium-ion (Na^+^)-pumping rhodopsin enabled the mutated version to bind carotenoid, while the wild-type version did not. Although the oligomeric assembly determines the function and stability of several rhodopsins,^26−29^ the effect of oligomer formation on the thermal stability of the protein and the effect of carotenoid binding on the thermal stability are not yet completely well-understood.

Here, we report the binding of the carotenoid Sal to a novel Na^+^-pumping rhodopsin from *Nonlabens (Donghaeana) dokdonensis* (DDR2) to form a thermally stable rhodopsin–carotenoid complex.^30−32^ There is no report in the literature whether this species naturally binds carotenoid. We show that the oligomerization process of DDR2 depends on its concentration and pH, which in turn affects the protein thermal stability. Furthermore, our studies revealed that the thermal stability of DDR2 is affected by Sal binding. Sal shows a very weak band in the CD spectrum due to lack of chirality, while a well-structured bisignate CD spectrum of DDR2–Sal complexes indicates an induced chirality of Sal following binding to DDR2.

## Experimental Section

### Expression, Purification, and Sample Preparation of DDR2

The protein (DDR2) was expressed as a recombinant protein in *Escherichia coli* and purified following the procedure described elsewhere.^30^ The protocol for the preparation of DDR2 has been described briefly in the Supporting Information (SI). Salinixanthin was extracted from xanthorhodopsin (xR) using the procedure described in refs (20) and (33). The artificial pigments were prepared by incubation of the apo-protein of DDR2 with 1.5 equiv of the appropriate synthetic retinal analogues at room temperature for 12–18 h. Otherwise stated explicitly, all of the experiments were done in an aqueous solution of neutral pH containing 0.06% *n*-dodecyl β-d-maltoside (DDM) and 300 mM NaCl. Suitable buffer solutions (final buffer concentration of 50 mM) were used to maintain the pH of the solution whenever required.

### Spectroscopic Studies

UV–visible absorption spectra of the protein samples were recorded using an Agilent 4583 diode-array spectrophotometer (Agilent Technologies, Palo Alto, CA), which was equipped with an Agilent 89090A thermostated cuvette holder as the temperature controller. The cuvette holder and the sample were equilibrated for 5 min at the desired temperature (to achieve a homogeneous heated sample) followed by monitoring the denaturation process. To evaluate the protein fraction that was denaturated, the sample was brought to 25 °C and centrifuged and the absorption spectrum was monitored. Scattering arising due to protein denaturation was reduced using 10% glycerol, and the baseline was corrected manually. CD spectra were recorded using a Chirascan CD spectrometer (Applied Photophysics), in which a temperature controller was attached. Size-exclusion chromatographic (SEC) analyses were performed in an AKTA FPLC system. Differential scanning fluorimetry (DSF) experiments were performed using a nano-DSF instrument (Prometheus NT.48).

#### Molecular Modeling

The crystal structure of DDR2 is still not available. Therefore, we have generated the three-dimensional structure of the protein using homology modeling in the Swiss-model server.^34,35^ Since DDR2 has more than 78% identity in the amino acid sequence with another sodium-ion-pumping rhodopsin *K. eikastus* rhodopsin 2 (KR2), and the crystal structures of the latter as a monomer and a pentamer are available, we have used the KR2 (PDBID: 6RF6 and 6REW for the monomer and pentamer, respectively, as templates for building the structural models of DDR2.

## Results and Discussion

### Thermal Stability of DDR2

**Figure 1:**
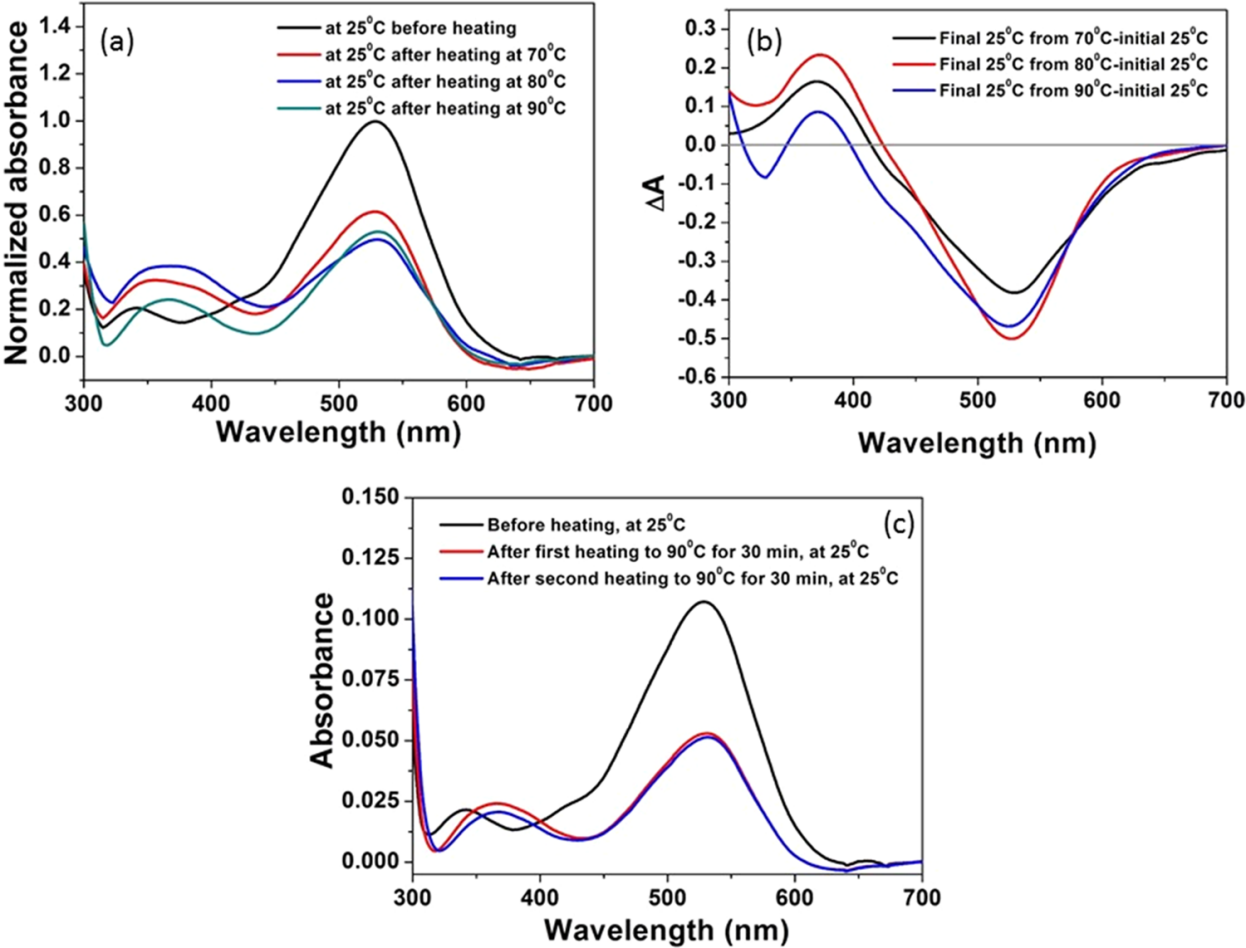
(a) Absorption spectra change of DDR2 (2 × 10^–5^ M) at 25 °C before and after heating to 70 and 80 °C for 10 min and to 90 °C for 30 min at pH 8. (b) Difference spectra obtained by subtracting the spectra after heating by that monitored at initial 25 °C. (c) Thermal stability of the heat-treated DDR2 (2 × 10^–6^ M) sample, prepared by heating it for 30 min at 90 °C, pH 8, followed by cooling down to 25 °C. The sample was heated to 90 °C for 30 min in two cycles, and the scattering-corrected absorption spectra at 25 °C after cooling are presented.

Studies of thermal stability of microbial rhodopsins have gained attention due to their applications in bioelectronic devices and optogenetics.^36,37^ We have employed several spectroscopic studies to investigate the thermal stability of DDR2. At room temperature and pH 8, DDR2 shows an absorption band with a maximum (λ_max_) at 528 nm (Figure 1a), originating from the absorption of the all-*trans* retinal chromophore covalently bound to the protein. Upon temperature increase to 55 °C, the λ_max_ of DDR2 was blue-shifted to 521 nm. Further warming the protein (2 × 10^–6^ M, pH 8) to 70, 80 °C (10 min at each temperature), and 90 °C (30 min) led to partial depletion of the absorption at 528 nm and the formation of a new band around 380 nm (Figure 1a,b). It was previously reported that during denaturation of microbial rhodopsins the retinal PSB is hydrolyzed and a retinal band with the maximum at around 377 nm appears.^33^ Therefore, the formation of a 380 nm band of DDR2 due to heating suggests that a fraction of the protein was denaturated once the protein was heated to above 70 °C. To ensure that the fraction was denatured due to heating, the samples were cooled to 25 °C, and their absorption was compared to the initial sample absorption. It is evident that the absorption band reached its original maximum at 528 nm, but a fraction of the pigment was denatured. Most of the denaturation took place at 70 °C and reached saturation at 80 °C (Figure 1a,b). About 60% of the protein was denatured at 80 °C, while the other fraction was stable even at 90 °C and probably experienced denaturation only at higher (>90 °C) temperatures. The increased intensity of the 380 nm band following temperature increase from 70 to 90 °C through 80 °C indicates denaturation of a higher fraction of the protein. The different thermal stability suggests that DDR2 consists of at least two different species, which are nonidentical in their thermal stability. The nature of these species will be discussed below. The fraction of protein that persists after heating to 90 °C for 30 min at pH 8, followed by cooling to 25 °C, shows high resistance to temperature. Upon reheating this fraction to 90 °C for 30 min, the denaturation of the protein is negligible (Figure 1c). This observation further supports the fact that the DDR2 protein consists of more than one form.

To shed light on the rationale underlying the heterogeneity of DDR2 thermal stability, we have studied the effect of concentration and pH on thermal stability. The percentage of DDR2 denaturation measured at two different concentrations, in which one is 30 times more concentrated and at different pH, due to heating at 80 °C for 30 min (Table S1) clearly revealed that the thermal stability of DDR2 is indeed concentration-dependent and was also affected by the pH value. The change in the absorption intensity due to heating at a high concentration (6 × 10^–5^ M) of DDR2 (Figure S1) indicates that the fraction of denaturation of DDR2 is notably decreased due to the protein concentration increasing. The concentration dependence indicates that the denaturation process may be associated with protein aggregation. It was previously shown that several microbial retinal proteins adopt an oligomeric form (mostly trimer or pentamer) at room temperature and neutral pH. Some of them are converted to their monomeric form due to heating, lowering the pH value, or change in the medium environment. For example, solubilization of the trimeric form of bacteriorhodopsin (BR) in Triton X-100 leads to formation of a monomeric form. This transformation is reflected by a blue-shifted absorption from 568 to 553 nm.^38^ TR showed as well a blue-shifted absorption from 530 nm to 525 nm due to the trimer–monomer transition obtained by heating above 73 °C.^33^ It is plausible that DDR2 is composed of a mixture of two oligomers in which one is unstable above 70 °C, whereas the other is stable even at 90 °C. The ratio between the two forms is concentration and pH dependent. The fraction of monomers is increased at low concentrations and low pH values.

To shed further light on the possible oligomeric forms of the protein, we have recorded the temperature-, concentration-, and pH-dependent CD spectra of DDR2 (Figure S2). The CD spectra of DDR2 show a positive distinct band at 543 nm (a red-shifted band relative to DDR2 absorption), whereas a negative band is not clearly observed. Similarly, the CD spectrum of the Na^+^-ion pumping rhodopsin KR2 in a DDM solution showed a positive band at 544 nm with an obscure negative band at around 450 nm.^28^ Upon heating at 80 °C for 5 min, the positive band of DDR2 was blue-shifted by 8 nm, while recooling to 25 °C reverted the band to 543 nm with an intensity decrease. The intensity decrease is due to the partial denaturation process caused by heating as observed in the absorption spectral studies described above. We have further checked the absorption and CD spectra at different pH values. The CD band maximum of DDR2 was red-shifted due to a change in the pH value from 9 to 4 (Figure S3). The red shift due to this pH change in the higher-concentration (2 × 10^–5^ M) sample from 545 to 563 nm is larger than that monitored at the low-concentration sample (2 × 10^–6^ M), which showed a change from 542 to 550 nm. We have compared the absorption change of DDR2 due to the pH decrease from 9 to 4 at the aforementioned concentrations (Figure S4). The higher-concentration (2 × 10^–5^ M) sample showed an absorption change from 528 nm at pH 9 to 551 nm at pH 4, similarly to the lower-concentration sample (2 × 10^–6^ M), which showed a red shift from 527 nm at pH 9 to 553 nm at pH 4. Namely, the absorption spectrum is not affected by the oligomerization form. In contrast, the CD spectrum is affected by the oligomerization form since the spectrum is modified by the excitonic interaction, which is due to protein oligomerization.

Analysis of pH-titration absorption spectra (Figure S5) of DDR2 using a modified Henderson–Hesselbach equation^20^ revealed a p*K*_a_ transition of 5.5, which we attribute to the p*K*_a_ of the PSB counterion. Besides this prominent transition, an additional small transition at about pH 7.2 is detected. In analogy to BR, we propose that it is associated with a titration of a protein residue that modifies the p*K*_a_ of the counterion.^39,40^ We have also studied the concentration dependence of the CD spectra of DDR2 at higher concentrations (6 × 10^–5^ to 5 × 10^–4^ M) at room temperature and pH 8 (Figure S6a). It was found that the CD spectrum experiences a significant change and the maximum of the positive CD band was gradually red-shifted as the concentration was increased from 6 × 10^–5^ to 5 × 10^–4^ M, accompanied by a formation of a negative band at 520 nm. We attribute this new CD spectrum to the excitonic interaction between retinal chromophores, which takes place at a concentration of above 4 × 10^–5^ M.

The CD spectrum of the KR2 protein, which adopts a pentameric form, is very similar to the spectrum of the presently studied DDR2 protein and has only a positive band and lacks a clear negative band.^28^ Recently, a theoretical study^41^ proposed that the CD spectrum of KR2 is dominated by Coulomb interaction between the retinal chromophores and negligible contributions of exchange and charge-transfer interactions to the excitonic coupling energy. Since DDR2 exhibits high similarity to KR2, it is plausible that a similar explanation can hold for the DDR2 pentameric form. However, we propose that at high concentrations, DDR2 pentamers aggregate and interact with each other such that the CD spectrum is completely changed, and a conventional spectrum with a negative and positive band dominates, which reflects an excitonic coupling between the retinal chromophores.

To further explore the possibility of monomer–oligomer transition, we have employed size-exclusion chromatography (SEC) and studied the DDR2 at different pH values. Figure S7 shows that the retention volumes at pH 9 or 7 are different from those of pH 3. Using a gel filtration molecular weight marker containing soluble proteins, we estimated the molecular weight of the fractions with elution volumes of 11.79, 10.84, and 9.47 mL as 214, 386, and 630 kDa, respectively. Previously, the SEC technique was used to study the pH-dependent oligomerization of GR.^42^ The change in the retention volume indicated that the protein adopts a monomeric form at pH 3 and an oligomeric form at pH 8. In addition, it was reported that the bisignate CD spectra of TR and BR are lost due to formation of a monomeric form.^33,38,43^ The change in the retention volume of DDR2 and also the loss of intensity in the CD spectra due to lowering of pH of the medium indicate that the oligomeric assemblies of DDR2 at pH 7 and 9 are different from those at pH 3. At pH 4, the SEC of the protein shows two distinct forms, each matching those of pH 7 or 9 and 3. As the size of the DDM-solubilized proteins depends on the surface area accessible to DDM, the size of the protein obtained through SEC could vary notably.^43^ For example, TR and *Natronomonas pharaonis* HR (NpHR) have molecular masses of 29.2 and 32.4 kDa, while the molecular weights of their DDM-soluble trimeric forms were reported to be 207.7 and 177 kDa, respectively. The apparent molecular weights of pentameric and monomeric KR2 were reported to be 250.3 and 131.4 kDa, respectively.^44^ In analogy with KR2, it is possible that the apparent molecular weights detected by us for DDR2 of 214, 386, and 630 kDa are associated with the monomer, pentamer, and dimer of pentamers forms, respectively. It was demonstrated^44^ that due to removal of sodium ions from the solution, the DDM-solubilized KR2 turns into a monomer. As mentioned earlier, DDR2 is structurally quite similar to KR2, and we propose that the thermal stability of the DDR2 monomer is lower than that of its pentameric form. Therefore, we have compared the thermal stability of DDR2 in 0.06% DDM solution with 300 mM NaCl and without any salt. It was found that DDR2 was thermally unstable in the absence of salt and completely denatured at 70 °C (Figure S6b). This result also supports our proposal that the second (presumably monomeric) form of DDR2 is less stable than its pentameric counterpart.

**Figure 2:**
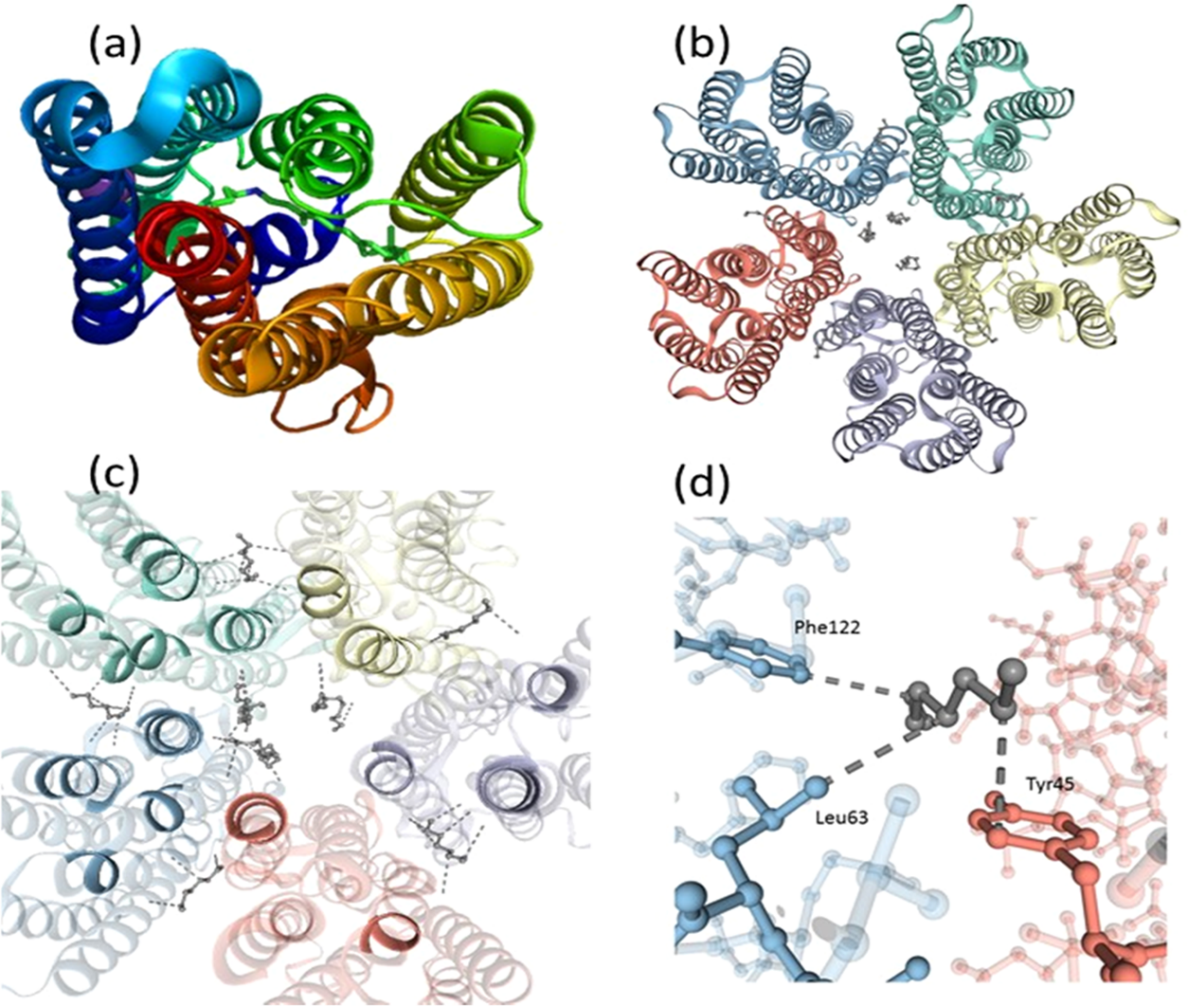
Three-dimensional (3-D) model structure of the DDR2 (a) monomer and (b) pentamer obtained through homology modeling in the Swis-model server. Crystal structures of KR2 (PDBID: 6RF6 and 6REW for monomer and pentamer, respectively) are used as templates. (c) Interactions, which also involve lipid molecules arising from the template protein, among the individual monomers in the pentameric assembly of the protein. (d) Enlarged view of one of the hydrophobic interactions shown in panel (c).

As described above, at pH 7, the protein fraction that is stable up to 90 °C is not converted to the thermally unstable monomeric form at room temperature, while it is converted to the monomeric form at low pH. TR is reported to adopt a monomeric form at pH 3 (below its p*K*_a_ of the primary counterion, Asp95) from its trimeric form at pH 7 at room temperature.^43^ In addition to the His61–Asp95 interactions, intra- and intermolecular interactions were proposed to be responsible for maintaining the trimeric assembly of the protein. Similar interactions can control the oligomerization equilibrium in DDR2. Therefore, due to protonation of protein residues, the pentamer structure became stable and the equilibrium of monomer–pentamer oligomerization shifted toward the monomeric form. The energy barrier for the transition is low, and therefore, it takes place at pH 3 at room temperature. In contrast, the fraction of the monomeric form does not change at pH 7 even after its denaturation, probably since the equilibrium with the pentameric form does not change following the monomeric denaturation or since the energy barrier for the transition is too high. The crystal structure of DDR2 is not known yet, and the protein has a large similarity in the amino acid sequence to the sodium-ion-pumping rhodopsin KR2. KR2 is reported to adopt both monomeric and pentameric forms, depending on the pH of the medium.^44−46^ A possible three-dimensional structure of DDR2 as the monomer and pentamer, obtained through homology modeling, is shown in Figure 2a,b, respectively.

The interactions between the different rotamers in the pentameric form of KR2 were reported based on the crystal structure.^44−47^ It was shown that the oligomerization of KR2 is mediated through hydrophobic interactions of the amino acids at the cytoplasmic and extracellular sides, while several direct and water-mediated hydrogen bonds make the interface. Our homology modeling of DDR2 revealed possible interactions between the different monomeric units (Figure 2c,d). The KR2 monomer was prepared through a single-point mutation (H30L, H30K as well as Y154F).^45^ It was found that the H30L mutant was notably unstable even at room temperature. Similarly, the monomeric form of GR was less stable than its trimeric counterpart.^26^ Therefore, it is conceivable that the interactions between the different monomeric units thermally stabilize the DDR2 oligomer relative to the monomeric form.

**Figure 3:**
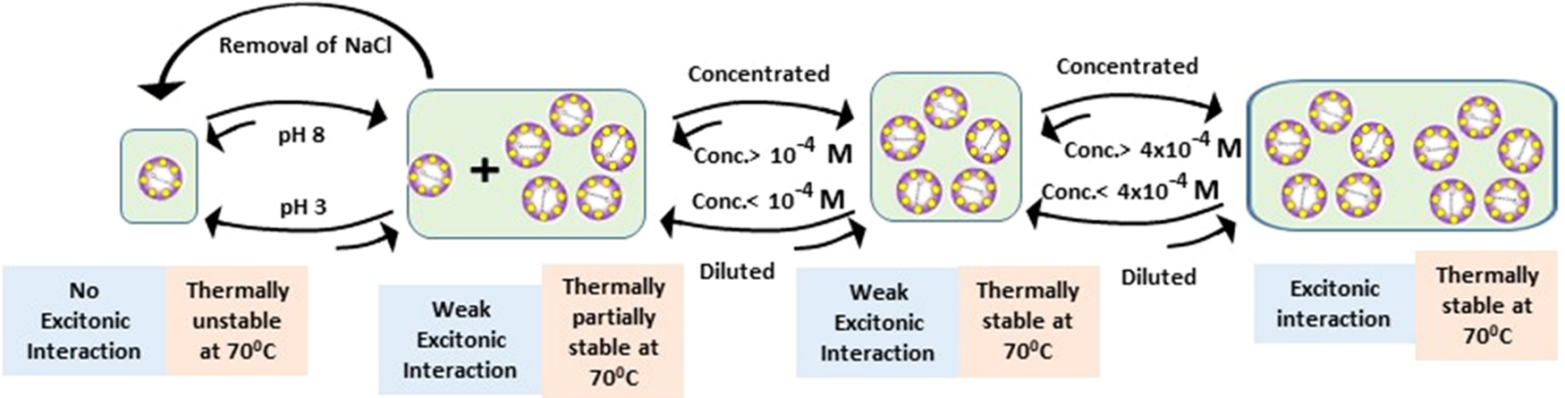
Schematic representation of oligomerization and thermal stability of DDR2 due to the change in concentration and pH of the sample. At moderate concentrations (4 × 10^–5^ to 2 × 10^–4^ M), the protein mostly remains as a pentamer, a fraction that is converted to a thermally less stable oligomer (presumably monomer) either due to dilution or due to lowering the pH to 3. The ratio of these two oligomers is dependent on the concentration of the sample. At very high concentrations, the CD spectral studies indicate an interaction among the pentamers of the protein. At this high concentration, the fraction of the thermally less stable oligomer is further decreased as the equilibrium is further shifted toward the oligomer of the pentamer form.

The above studies indicate that the protein DDR2 adopts different aggregated forms, which have nonidentical thermal stability. One of them (presumably the monomer) is the thermally less stable one, whereas the pentamer and its aggregated forms show increased thermal stability. The fraction of each form is affected by the protein concentration as well as by the pH value and the presence of salt. The formation of the monomeric form is decreased as the concentration of the sample is increased and the pH is raised above 5. The proposed effect of concentration and pH on the oligomerization and thermal stability of DDR2 is summarized in Figure 3.

### Retinal–Salinixanthin Interaction in DDR2

**Figure 4:**
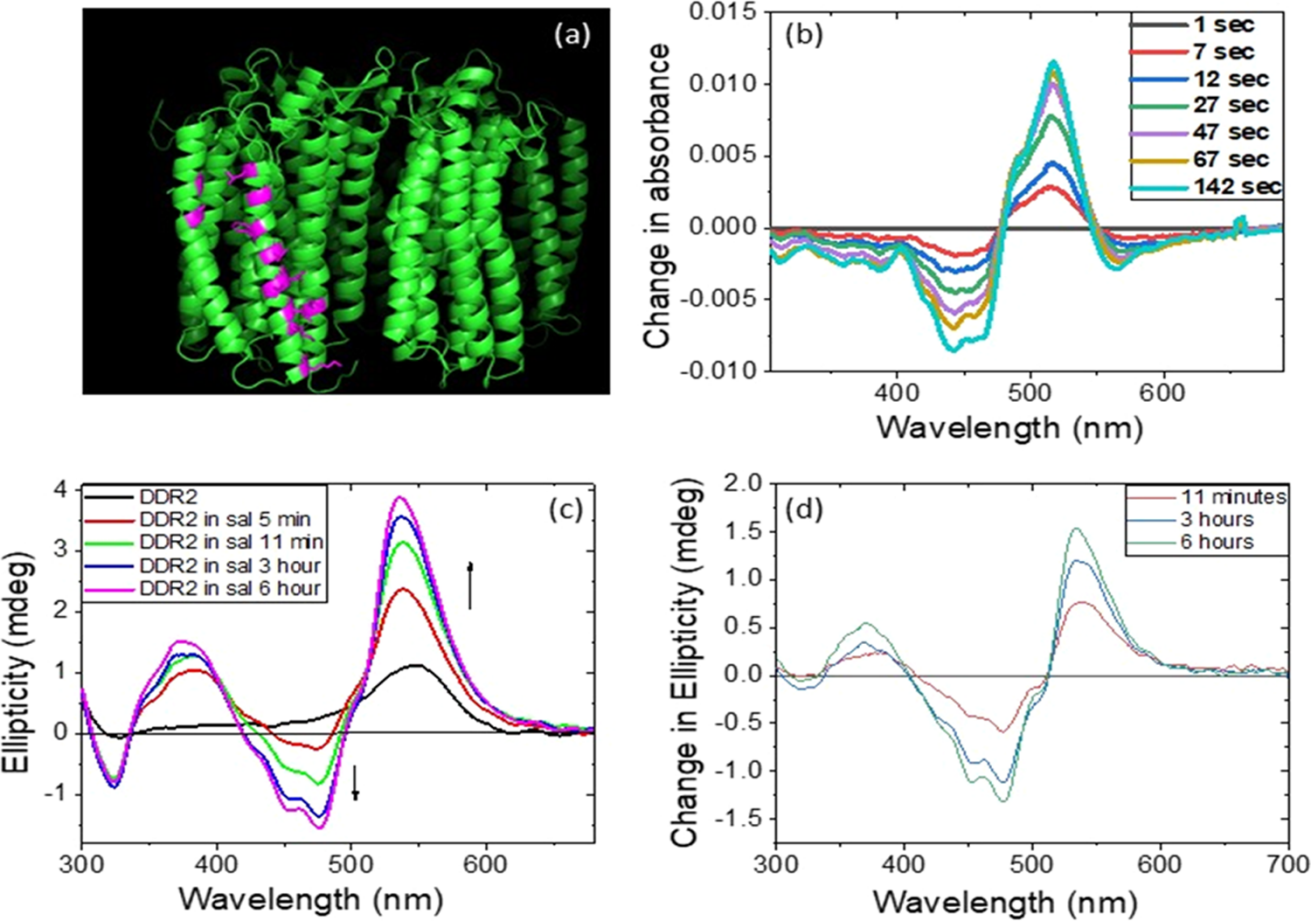
(a) Carotenoid binding site of DDR2, as obtained from the homology modeling (see Figure S8 for the amino acid sequence in the carotenoid binding site). (b) CD difference spectra obtained by subtracting the spectra recorded at longer times (7, 12, 27, 47, 67, and 142 s) by the initial spectrum. (c) CD spectra of DDR2 as well as those of DDR2–Sal mixtures over time. (d) Difference spectra obtained by subtracting the 11 min, 3 h, and 6 h CD spectra by that recorded after 5 min.

Out of the vast number of known microbial rhodopsins, only a few are known to bind carotenoids. It was suggested earlier that the presence of the tryptophan group (Trp138) in the retinal binding pocket restricts carotenoid binding to bacteriorhodopsin (BR).^46^ The presence of a comparatively smaller glycine residue facilitates binding of the carotenoid Sal to GR, xR, and TR.^14,19,20,33^ The sequence analysis of DDR2 and its 3-D structure obtained from homology modeling reveal that the glycine residue (Gly171) is located similarly to xR and may allow Sal binding (Figure S8). Homology modeling revealed as well that a potential carotenoid binding cavity exists in DDR2 as suggested for KR2 (Figure 4a). Specifically, out of the 12 amino acid residues present in the vicinity of the retinal binding pocket of DDR2, nine of them match those of KR2 (Figure S8). The presence of a smaller glycine residue was reported as well in KR2; however, binding of carotenoid was not reported. This possibility is still open for future studies.

We have checked the possible binding of carotenoid to DDR2 by incubating DDR2 with 1 equiv of salinixanthin (Sal). The binding process was monitored by absorption and CD spectroscopies. The changes in absorption spectra of DDR2 at different time intervals are shown in Figure S9. Sal, a C40 carotenoid, shows an absorption maximum at 485 nm with two shoulders at around 460 and 512 nm. It was previously reported that the carotenoid vibronic absorption bands become narrow due to reconstitution of GR with Sal.^19,47^ Similar vibronic band alteration was reported for the binding of Sal to xR.^14^ However, no well-resolved vibronic bands of the carotenoid were observed in the absorption spectra of the TR–Sal complex.^21^ Addition of Sal to DDR2 induced a change in the absorption spectrum of DDR2, but a well-resolved vibronic band like in xR or GR–Sal complexes was not observed (Figure S9). We have analyzed the difference spectra (Figure 4b) obtained by subtracting the initial spectrum from that recorded at 7, 12, 27, 47, 67, and 142 s. The difference spectra show changes in absorption at 520 and 445 nm over time due to the addition of Sal to DDR2. A similar behavior was observed due to addition of Sal to TR, and it was shown that the behavior is different from that obtained due to solubilization of Sal in DDM micelles. Therefore, the change in absorption spectra of the DDR2–Sal mixture indicates the specific interaction of Sal with DDR2. To gain further insights into the interaction between Sal and DDR2, we have recorded the CD spectra of DDR2, Sal, and their complexes (Figure 4c). As reported earlier,^33^ Sal shows a weak CD spectrum due to lack of Sal chirality in solution, and the CD spectrum of DDR2 is feeble as well. However, a clear bisignate CD spectrum with positive and negative bands at 536 and 475 nm, respectively, accompanied by a clear vibronic structure is monitored following addition of 1 equiv of Sal to DDR2. The difference spectra obtained by subtracting the initial spectrum recorded after 5 min of incubation from the spectra recorded at 11 min, 3 h, and 6 h show formation of negative and positive CD bands at 540 and 475 nm, respectively (Figure 4d). The difference spectra indicate that the binding of Sal to DDR2 is not instantaneous, and the binding process requires hours. The binding process of Sal to DDR2 can be accelerated by heating, which is described in the following section. In addition, SEC experiments on the protein–Sal complex indicated that the oligomeric form of the protein is not affected by the binding of Sal (Figure S7). To understand the ratio of the carotenoid that binds to the protein, we have measured the CD spectra of DDR2–Sal mixtures with varying Sal concentrations (0.6, 1.2, and 2 concentration equivalent). It was observed that the CD intensity of the DDR2–Sal mixture saturates at slightly above a 1:1 protein/Sal ratio (Figure S10), indicating that one carotenoid binds to one protein molecule. Efficient energy transfer between Sal and the retinal chromophore was detected for xR.^48^ We could not detect a similar efficient energy transfer for DDR2 using fluorescence spectroscopy and the excitation spectrum. It is possible that although binding of Sal occurs, the dihedral angle between the two chromophores (retinal and Sal) is not appropriate for energy transfer. It may indicate that Sal is not the native carotenoid that binds to DDR2. Further studies should clarify the structure of the native carotenoid.

The underlying origin of the bisignate CD spectrum of the first reported rhodopsin–carotenoid complex protein xR is debated. It was proposed^49^ that two factors affect the unique CD spectrum of xR: (i) excitonic coupling between the retinal and carotenoid in a single xR molecule and (ii) induced chirality of Sal due to binding with the protein. More recently,^50^ the theoretical vibronic exciton model combined with a transition–density–fragment interaction method predicted that vibronic coupling between carotenoid and the retinal in xR is crucial for formation of the bisignate CD spectrum of xR. Different mechanisms were proposed,^51−53^ indicating that excitonic interaction among the Sal chromophores located at different protein subunits, as well as the retinal chirality contribute to the CD spectrum of xR. The CD spectrum of the TR (trimer)–Sal (1:1) complex at 25 °C and pH 7 showed two positive bands at 326 and 482 nm and two negative bands at 376 and 547 nm. It was reported that the CD spectrum of the TR(trimer)–Sal (1:1) complex is quite similar to that of the TR(monomer)–Sal (1:1) complex, indicating that the association of different Sal units does not play a role in affecting the CD spectrum of these complexes. In TR–Sal complexes,^21^ it was proposed that the 546 nm band has major contribution from the interaction between retinal and Sal, while the 485 nm band originates mainly due to the induced chirality of Sal.

**Figure 5:**
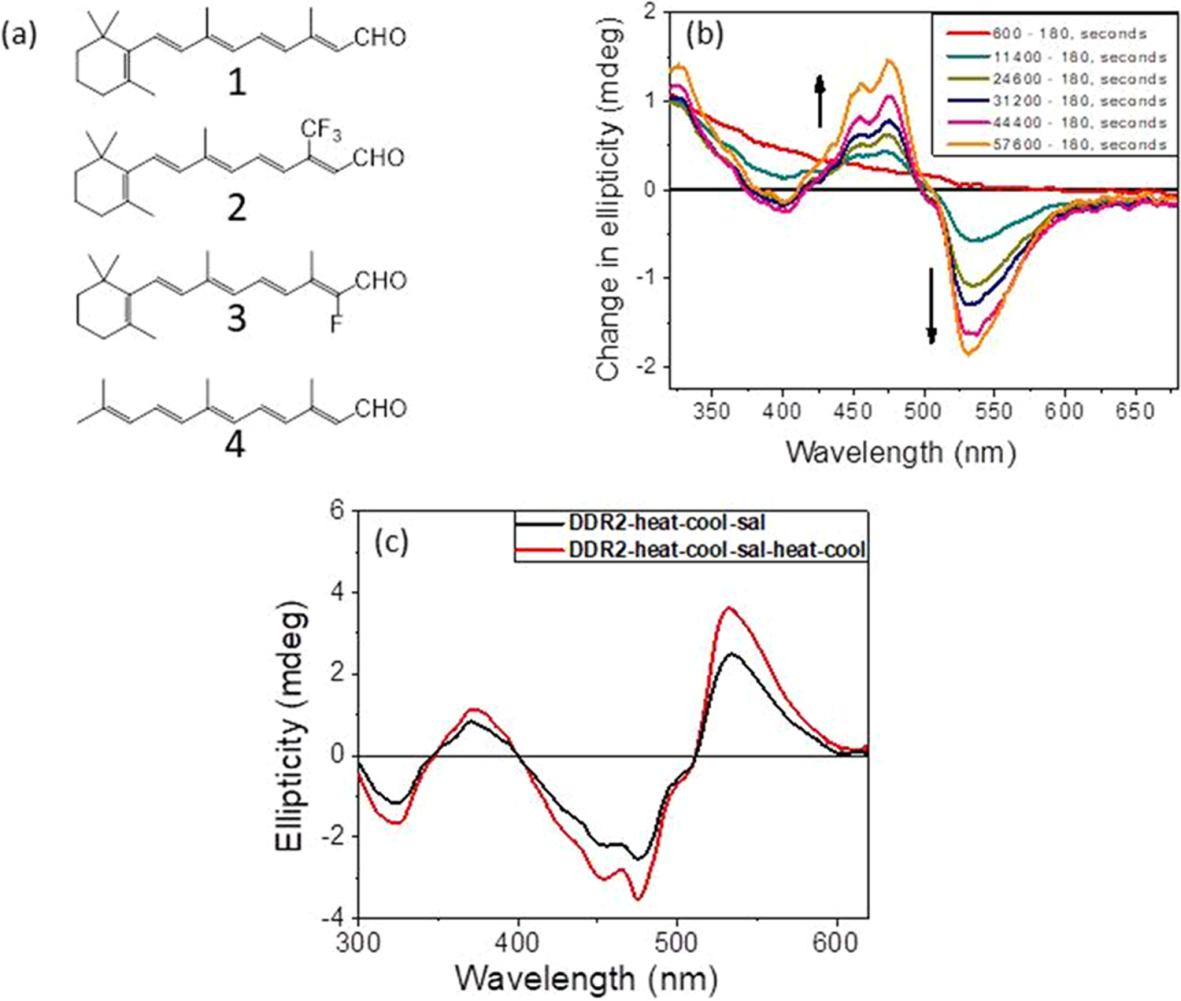
(a) Chemical structure of all-*trans* retinal and the modified synthetic retinals used for this study. (b) CD spectra of the DDR2 and Sal mixture, recorded at different time intervals. (c) CD spectra of the DDR2 sample obtained after heating at 80 °C for 30 min, followed by cooling at 25 °C and addition of Sal (black). Spectrum of the complex recorded after heating it to 80 °C, followed by cooling to 25 °C, is also shown (red).

To clarify the origin of the CD spectrum of the DDR2–Sal complex and especially if the positive band is associated with retinal chirality, we have studied the CD spectra of complexes formed between artificial DDR2 pigments derived from synthetic retinals and Sal (Figure S11). The CD spectra of the complexes formed between the artificial DDR2 pigments and Sal are shown in Figure 5b. DDR2 artificial pigments derived from 13–CF_3_ retinal (**2**) and 14-fluoro retinal (**3**) showed a red-shifted absorption band compared to the wild-type DDR2, while that derived from linear retinal (**4**) showed a blue-shifted absorption band (Figure S11). In keeping with these red-shifted absorption bands, the positive band at 536 nm of the CD spectra of the artificial pigments (**2** and **3**) was red-shifted, while the negative band at 475 nm remained almost unchanged. Therefore, we propose that the positive CD band of the DDR2–Sal complex has significant contribution from the retinal chromophore, while the negative band originates from the induced chirality of Sal. However, the CD bands of the complex formed between Sal and the artificial DDR2 pigment derived from the linear retinal (**4**) did not show significant change relative to native DDR2. Therefore, it is plausible that the apparent blue-shifted positive band of DDR2 (linear retinal–Sal complex) is masked by the strong negative band arising from the induced chirality of Sal.

### Effect of Temperature on DDR2–Sal Complexes

To understand the effect of temperature on the thermal stability of DDR2–Sal complexes, we have recorded the absorption and CD spectra of this complex at different temperatures (Figure 5c). The absorption spectra (Figure S11) revealed increased vibronic bands due to heating, but no significant increase in absorption at 380 nm was detected, indicating the stability of the pigment. Therefore, unlike DDR2 (2 × 10^–6^ M), which partially denatured due to heating at above 70 °C, the DDR2–Sal complex showed a comparatively higher resistance to temperature. The CD spectra also showed more prominent vibronic bands due to heating. Recently, an increased thermal stability of TR and Tara76 rhodopsin was detected following binding of a carotenoid.^24^ To further study the increased thermal stability of DDR2 due to binding of Sal, we carried out differential scanning fluorimetry (DSF) studies of DDR2 in the absence and presence of Sal (Figure S12). The ratio of the emission intensity at 330 nm to that at 350 nm of the tryptophan (Trp) residue was recorded between the 20 and 95 °C temperature range. The emission intensity of Trp is susceptible to the change in the environment, and it was reported that exposure to solvent increases the emission intensity.^54,55^ Therefore, this technique has been used to study the folding and denaturation of several proteins. The reported melting temperature (*T*_m_) is the midpoint of the transition. DSF studies revealed two transitions of DDR2 at pH 7 with *T*_m_ values of about 55 and 67 °C (Figure S12). Taking into account the absorption studies described above, it is plausible that the 55 °C transition is due to protein conformational alteration, whereas the second transition is due to denaturation of the thermally less stable oligomer of the protein. The denaturation transition of the pentamers is not detected since it is above the measured temperature.

In the DDR2–Sal complex, the first transition is absent, which may indicate that the protein conformation change of DDR2 is prevented in the presence of Sal. The DDR2 obtained by heating to 80 °C for 10 min, followed by cooling to 25 °C, does not show any transition at the studied temperature range (20–95 °C). Therefore, it can be concluded that binding of Sal induces increased protein thermal stability. The DSF study also supports the fact that the fraction of the protein that did not denature at 80 °C (10 min heating) is thermally stable, as indicated by the absorption studies.

The present studies revealed that at low concentrations DDR2 adopts a heterogeneous mixture of aggregated protein forms and consists of two fractions, which have different thermal stabilities. We propose that these two fractions represent two different oligomeric forms of the protein (monomeric and pentameric forms). At higher concentrations, the oligomeric form consists of pentamers and pentamer aggregation. This form is more thermally stable than the other (presumably monomeric) form. This conclusion is analogous to studies of GR,^23^ which compared the stability of trimeric and monomeric forms. It was proposed that the monomer of GR adopts a loosened structure and the Schiff base linkage is more easily bleached by a hydroxyl amine reaction or heating. In addition, it was shown that the presence of Sal affects the protein–retinal interactions in xR, and a faster binding of retinal to apo-membrane of xR in the presence of Sal was observed.^20^ Therefore, it is plausible that the higher thermal stability of DDR2 in the presence of Sal is due to the specific interaction between the retinal and the carotenoid, which restricts the hydrolysis of the PSB linkage. Future studies should clarify the mechanism by which Sal affects the protein thermal stability.

## Conclusions

The binding of carotenoid salinixanthin (Sal) with a novel sodium-ion (Na^+^)-pumping rhodopsin DDR2 is studied along with the effect on the oligomerization and thermal stability of the protein due to interaction with Sal. UV–Vis absorption and CD spectroscopic studies were used to monitor the carotenoid binding with the protein. Size-exclusion chromatography (SEC) and differential scanning fluorimetry (DSF) have been employed to understand the oligomerization and thermal stability of the protein in both the presence and absence of the carotenoid. Our studies revealed that in the concentration range of 2 × 10^–5^ to 1 × 10^–4^ M, DDR2 remains presumably in its pentameric form at room temperature and neutral pH, while temperature elevation converts a fraction of it to a thermally less stable oligomeric form. The oligomer-to-monomer ratio of these two forms was found to be dependent on the concentration and pH of the sample. CD spectral studies suggested the formation of pentamer aggregates at a very high concentration of the protein (5 × 10^–4^ M or above). Both DDR2 and Sal show a very weak CD spectrum due to lack of chirality, while binding of Sal to DDR2 shows a clear bisignate CD spectrum. Our studies revealed that the negative CD band originated due to induced chirality of Sal, while the positive CD band has contribution from chirality of both the retinal and the carotenoid. As the interaction of Sal with DDR2 leads to a thermally stable rhodopsin–carotenoid complex, it is expected to encourage further studies on its light-harvesting properties.

## Supporting Information Available

Method for expression and purification of the protein (DDR2); thermal stability of the protein at different concentrations; size-exclusion chromatography of the protein; absorption and circular dichroism studies of the protein–Sal mixtures; and results of DSF studies (PDF)
