# Supplementary material for: Retinal–Carotenoid Interactions in a Sodium-Ion-Pumping Rhodopsin: Implications on Oligomerization and Thermal Stability
Source: J Phys Chem B. 2023 Mar 1;127(10):2128–37. doi: 10.1021/acs.jpcb.2c07502 (PMC10026069; doi:10.1021/acs.jpcb.2c07502)
Supplement: Supplementary file 1 — jp2c07502_si_001.pdf [file jp2c07502_si_001.pdf]

Supporting information for

Retinal-Carotenoid Interactions in a Sodium Ion-Pumping Rhodopsin: Implications on Oligomerization and Thermal Stability

Mihir Ghosh[a†1], Ramprasad Misra[a†2], Sudeshna Bhattacharya,[a] Koushik Majhi,[a] Kwang-Hwan Jung[b], Mordechai Sheves[a*]

[a]*Department of Molecular Chemistry and Materials Science, Weizmann Institute of Science, Rehovot, 76100, Israel*

[b]*Department of Life Science and Institute of Biological Interfaces, Sogang University, Seoul, 04107, South Korea*

Method for Expression and Purification of DDR2

Codon optimized DDR2 was expressed in Luria–Bertani (LB) medium as recombinant protein in *Escherichia coli* BL21 (DE3) cells. *E. coli* transformants were grown in the presence of ampicillin (50 µg mL^{-1}) at 37 °C until the absorption intensity at 600 nm (λ_{600}) reaches to about 0.8. The cells were then induced with 0.1% isopropyl β-D-1-thiogalactopyranoside (IPTG) and 10 µM all-*trans* retinal for 8 hours. Pink-colored cells were harvested by centrifugation (7000 rpm) at 4 °C, followed by resuspension with buffer S (50 mM MeS, 300 mM NaCl, 5 mM imidazole, 5 mM $MgCl_2$; pH 6) that also contains 1% DDM (*n*-dodecyl-β-D-maltoside), lysozyme (0.1 mg mL^{-1}), DNAse and protease inhibitor. The resulting mixture was stirred overnight in the dark at room temperature. The extracted protein was collected as supernatant after centrifugation (18000 rpm) of the stirred solution at 4 °C for 30 min. The protein was purified using a Ni^{2+} NTA histidine-tagged agarose column. The column with histidine-tagged protein was washed with buffer W (50 mM MeS, 300 mM NaCl, 50 mM imidazole, 0.06% DDM; pH 6) and eluted with buffer E (0.06% DDM, 50 mM Tris–HCl, 300 mM NaCl, 50 mM HCl, 150 mM imidazole; pH 7.5). The eluted protein was washed using Amicon Ultra centrifugal filter devices and the washing was repeated for three times using a 0.02% DDM solution and 100 mM NaCl. The final DDR2 were kept at 4 °C in 0.06% DDM and 300 mM NaCl before use.

1. Present Address: Department of Chemistry, Kattankulathur Campus, SRM Institute of Science and Technology, Tamilnadu 603203, India
2. Present Address: Institute for Biology, Experimental Biophysics,
Humboldt-Universitat zu Berlin, Berlin 10115, Germany
† These authors contributed equally.
* Corresponding author: email address: mudi.sheves@weizmann.ac.il

Table S1: Comparison of percentage of denaturation due to heating of DDR2 at 80 ^{o}C for 30 minutes at high (6 x 10^{-5} M) and low concentration (2 x 10^{-6} M) at different pH of the media.

pH of the medium	Percentage (%) of denaturation of DDR2	
	High Conc. (6 x 10^{-5} M)	Low Conc. (2 x 10^{-6} M)
3	68	90
4	43	74
7	21	98
8	22	51
9	22	52
10	29	41

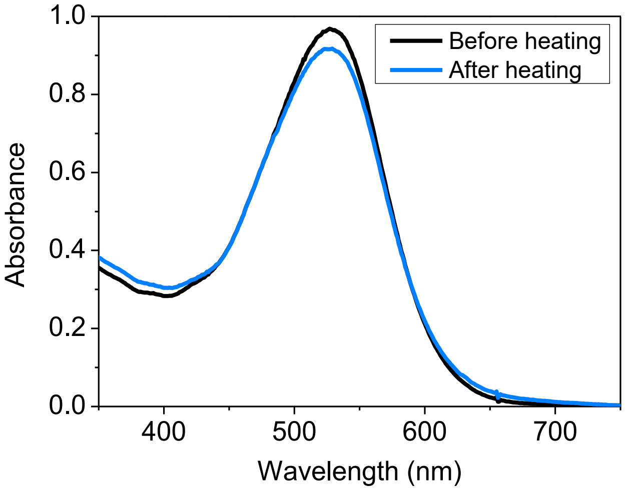

Figure S1: Change in absorption of DDR2 (2 x 10^{-5} M) at pH 8 due to heating from 25 ^{o}C to 80 ^{o}C for 30 minutes and cooling it back to 25 ^{o}C. Unlike the low concentration sample (2 x 10^{-6} M), the denaturation due to heating at this concentration is much lower.

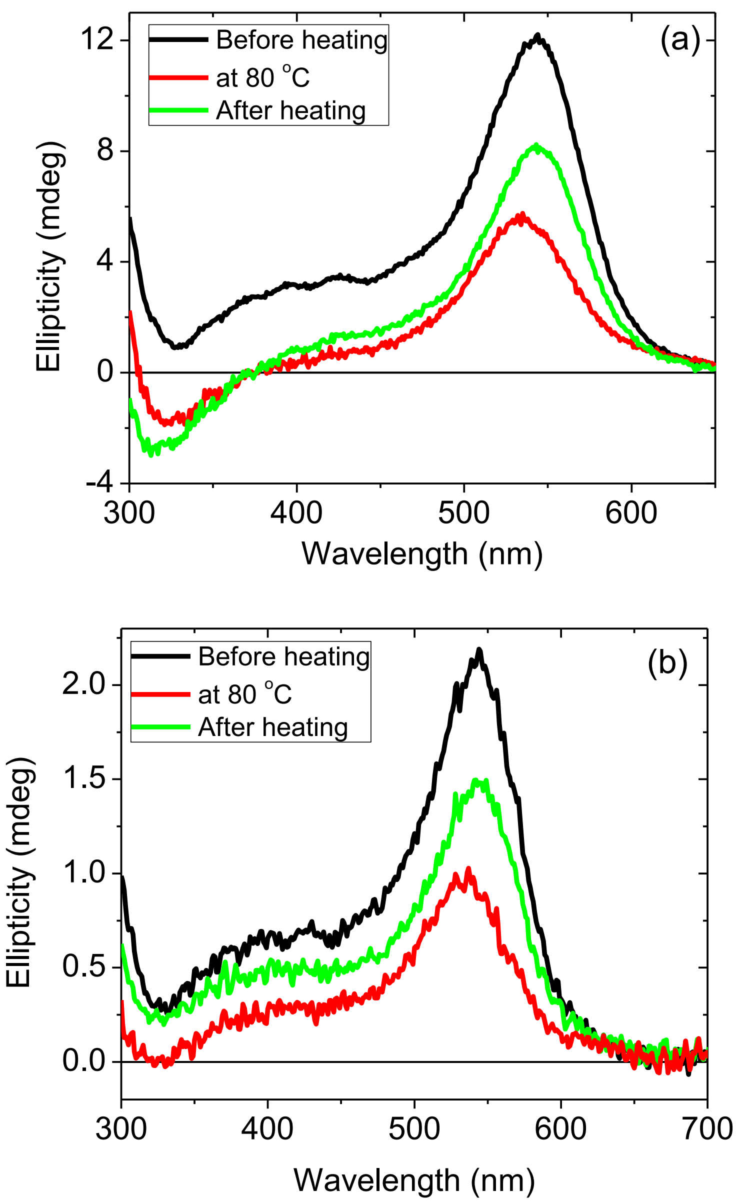

Figure S2: The temperature-dependent CD spectra of DDR2 at (a) high (2 x 10^{-5} M) and (b) low concentrations (2 x 10^{-6} M). The spectra recorded at 25 and 80 °C as well as those after cooling down the heated samples to 25 °C are shown.

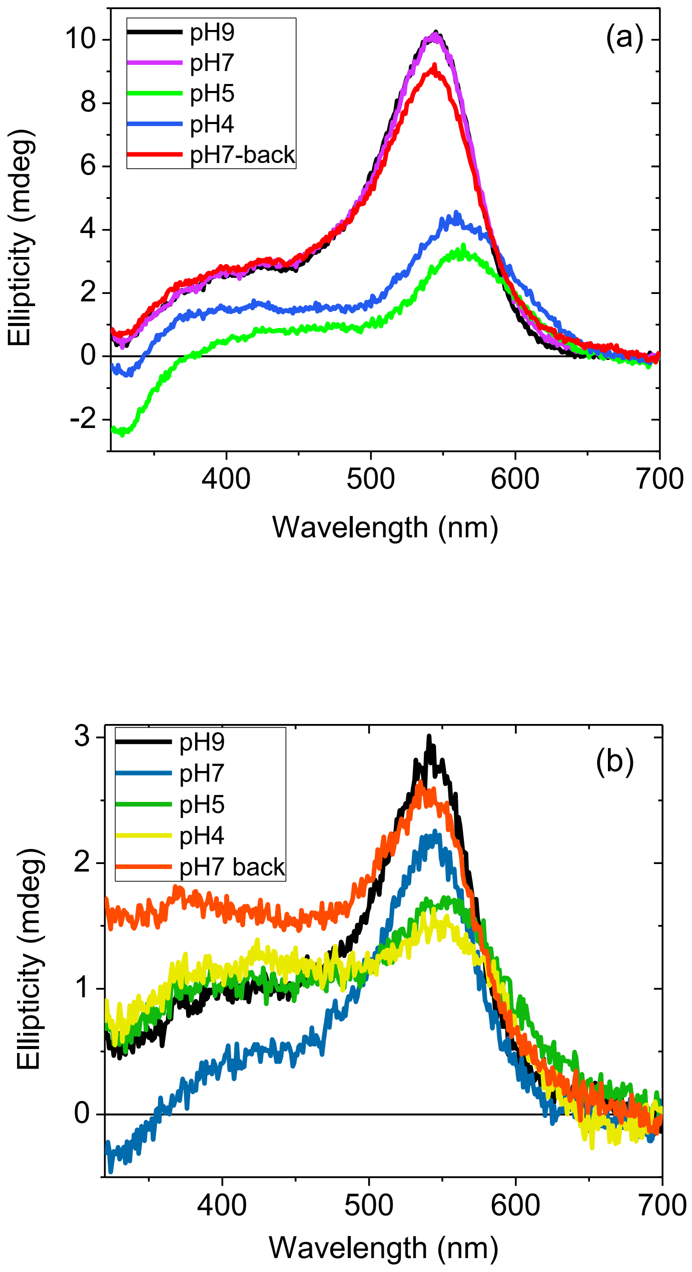

Figure S3: The pH-dependent CD spectrum of DDR2 at (a) high (2 x 10^{-5} M) and (b) low (2 x 10^{-6} M) concentrations. A red shift of the positive band was observed due to change in pH from 9 to 3. The red shift is greater in case of high concentration sample than its low concentration counterpart.

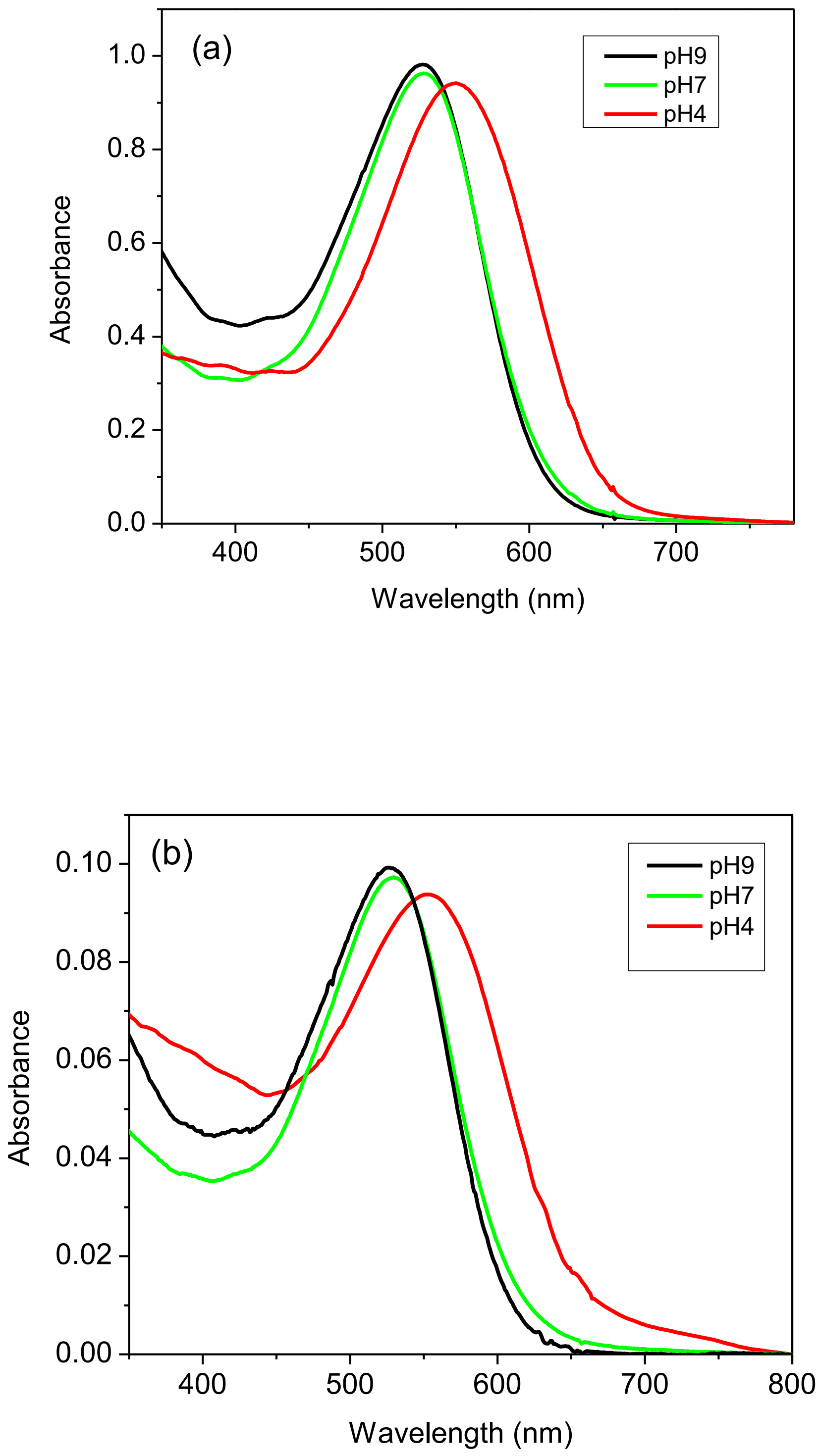

Figure S4: The change in absorption maxima of DDR2 due to change in pH from 9 to 4 through 7 at (a) high (2 x 10^{-5} M) and (b) low (2 x 10^{-6} M) concentrations.

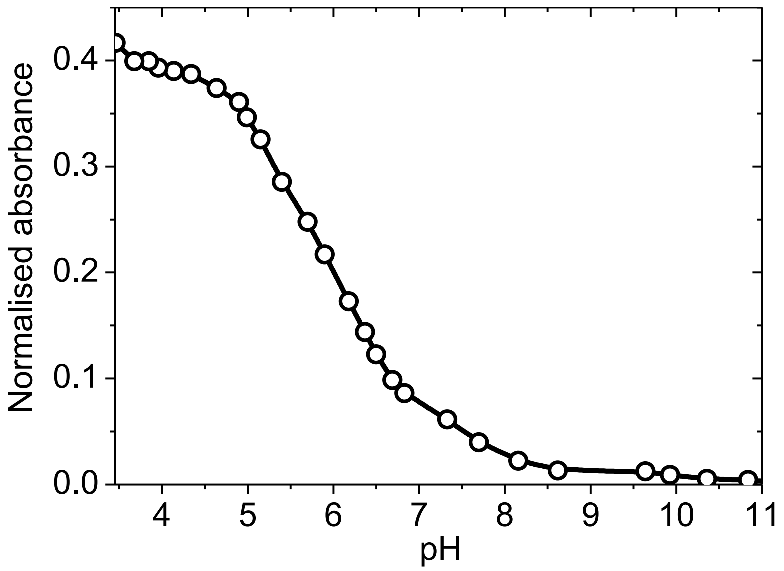

Figure S5: pH titration curve of DDR2 at concentration 6 X 10^{-5} M.

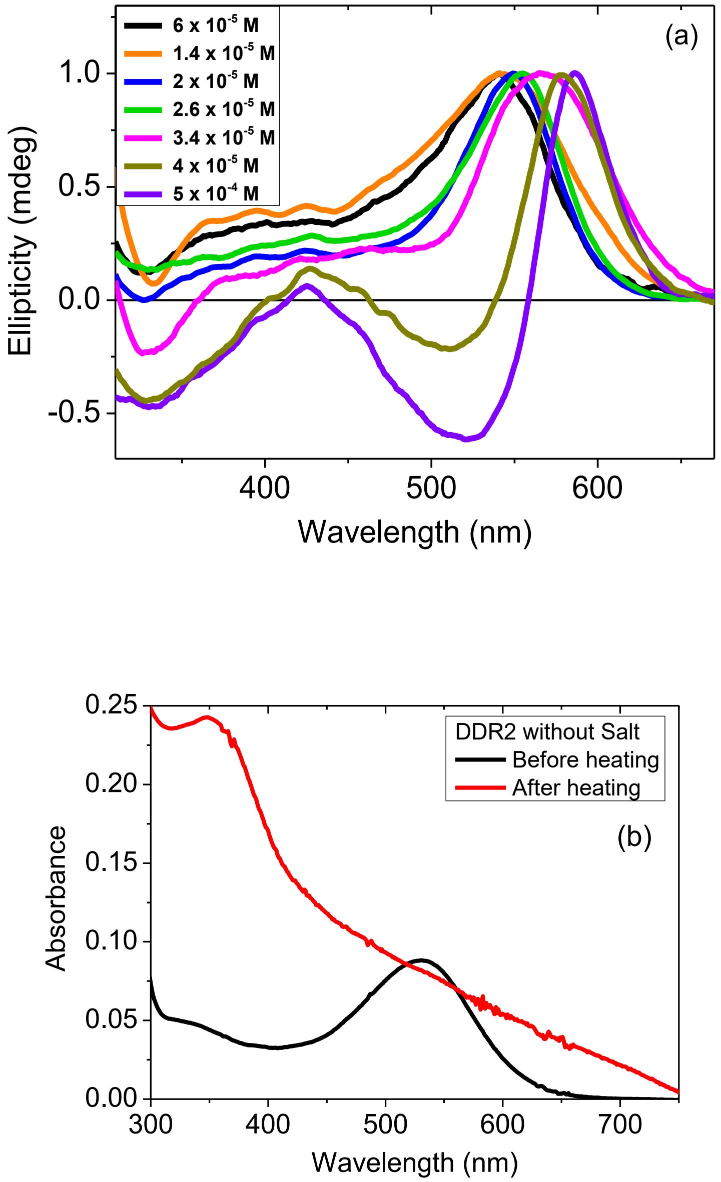

Figure S6: (a) CD spectra of DDR2 (normalized at the positive band) in pH8 in different concentrations (6 x 10^{-5} M to 5 x 10^{-4} M). A gradual red shift of retinal band is observed due to increase in concentration of the protein. (b) Thermal stability of DDM-solubilized DDR2 (without salt). The protein denatures completely within 35 minutes due to heating at 80 ^{o}C (red curve).

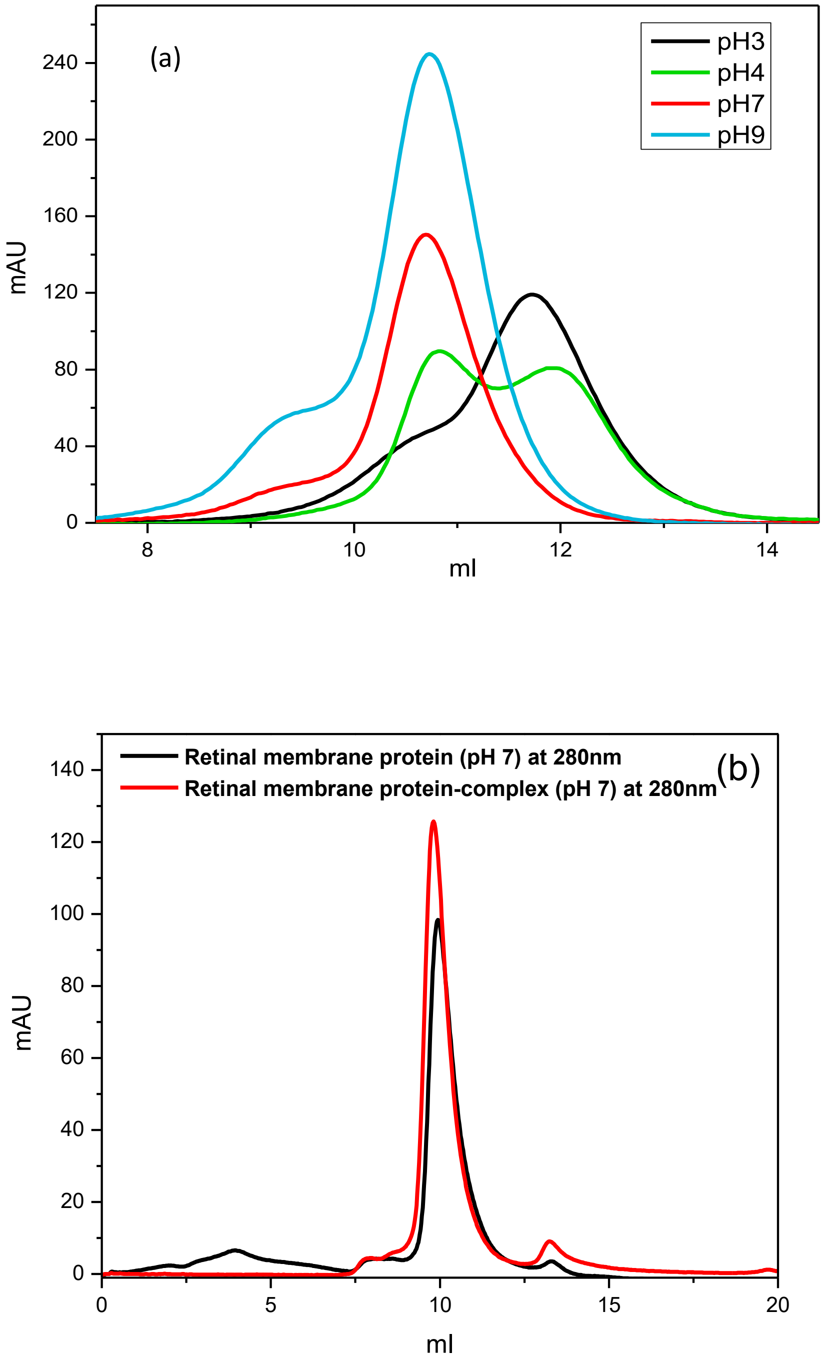

Figure S7: (a) The Size exclusion chromatogram of DDR2 at pH 3, 4, 7 and 9. The retention volume at pH 7 and 9 are than that of pH 3. At pH 4, the result indicates formation of two species of DDR2. (b) Size exclusion chromatogram of DDR2 in presence and absence of Sal.

```
XR            MLQELP------------TLTPGQYSLVFNMFSFTVATMTASFVFFVLARNNVAPKYRIS  48
DDR2          MIQDLGNSNFENYVGATDGFSEMAYQMTSHVLTLGYAVMFAGLLYFILTIKNVDKKYRMS  60
KR2           MTQELGNANFENFIGATEGFSEIAYQFTSHILTLGYAVMLAGLLYFILTIKNVDKKFQMS  60
              * *:*          .   ::   *.:. :::::  *.* *.:::*:*: :**  *:::*

XR            MMVSALVVFIAGYHYFRITSSWEAAYALQN----GMYQPTGELFNDAYRYVDWLLTVPLL  104
DDR2          NILSAVVMVSAALLLYAQAGNWTESFAFDAERGKYFLVEGGDLFNNGYRYLNWLIDVPML  120
KR2           NILSAVVMVSAFLLLYAQAQNWTSSFTFNEEVGRYFLDPSGDLFNNGYRYLNWLIDVPML  120
               ::**:*:. *    :  : .*  :::::      :    *:***:.***::**: **:*

XR            TVELVLVMGLPKNERGPLAAKLGFLAALMIVLGYPGEVSENAALFGTRGLWGFLSTIPFV  164
DDR2          LFQILFVVQLTKSKLSSVRNQFWFSGAMMIITGYIGQYYEVTDL-SAFFIWGAISTVFFF  179
KR2           LFQILFVVSLTTSKFSSVRNQFWFSGAMMIITGYIGQFYEVSNL-TAFLVWGAISSAFFF  179
               .::::*: * ..: . :  :: * .*:**: ** *:  * : *  :  :** :*:  *.

XR            WILYILFTQLGDTIQRQSSRVSTLLGNARLLLLATWGFYPIAYMIPMAFPEAFPSNTPGT  224
DDR2          HILWLMNKVIKEGKVGIPKKGQKILSNIWILFLVSWFLYPGAYLMPHLGGIEGFLFNESG  239
KR2           HILWVMKKVINEGKEGISPAGQKILSNIWILFLISWTLYPGAYLMPYLTGVDGFLYSEDG  239
               **::: . : :         ..:*.*  :*:* :* :** **::*          . .

XR            IVALQVGYTIADVLAKAGYGVLIYNIAKAKSEEEGFNVSEMVEPATASA     273
DDR2          VVGRQITYTIADVCSKVIYGVLLGNLALVLSKNKEMI-----ET-----     278
KR2           VMARQLVYTIADVSSKVIYGVLLGNLAITLSKNKELV-----EANS---     280
              ::. *: ****** :*. ****: *:* . *::: :      *
```

Figure S8: Comparison of amino acid sequence of DDR2 with KR2 and XR. The amino acids in the carotenoid binding site are marked in pink. In all these proteins, the Glycine residue (Gly171 for DDR2) is conserved in the retinal binding site.

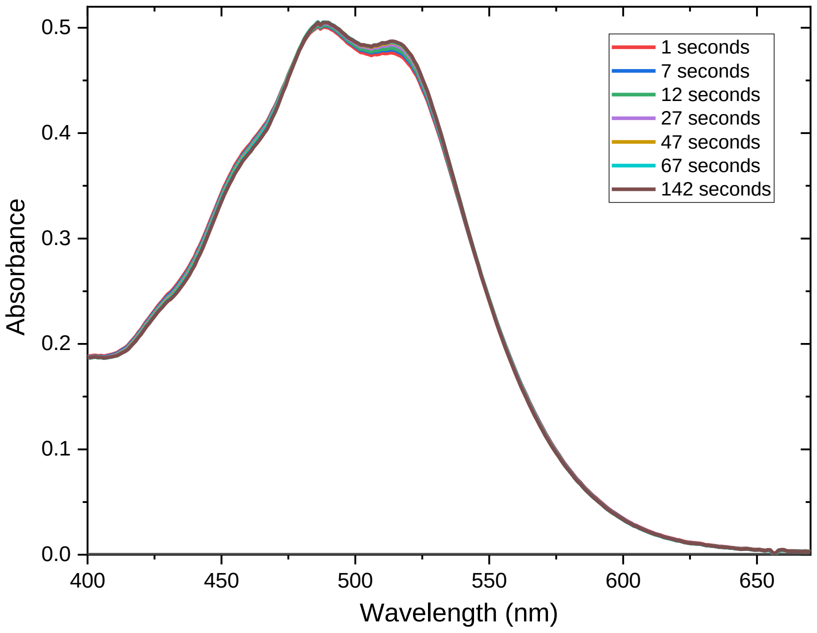

Figure S9: The change in absorption spectra of DDR2-Sal mixtures over time. Similarly to TR-Sal complexes, the change in absorption due to addition of Sal to DDR2 is smaller than that observed in GR-Sal complex and xR.

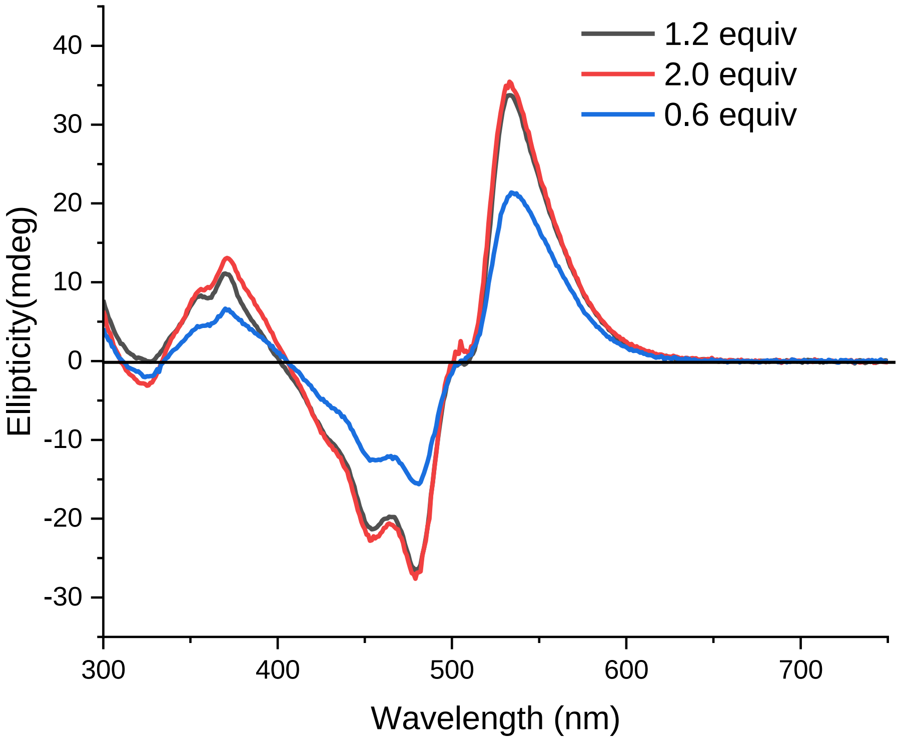

Figure S10: CD spectra of DDR2-Sal complexes with varying Sal equivalents (0.6, 1.2 and 2 equivalents).

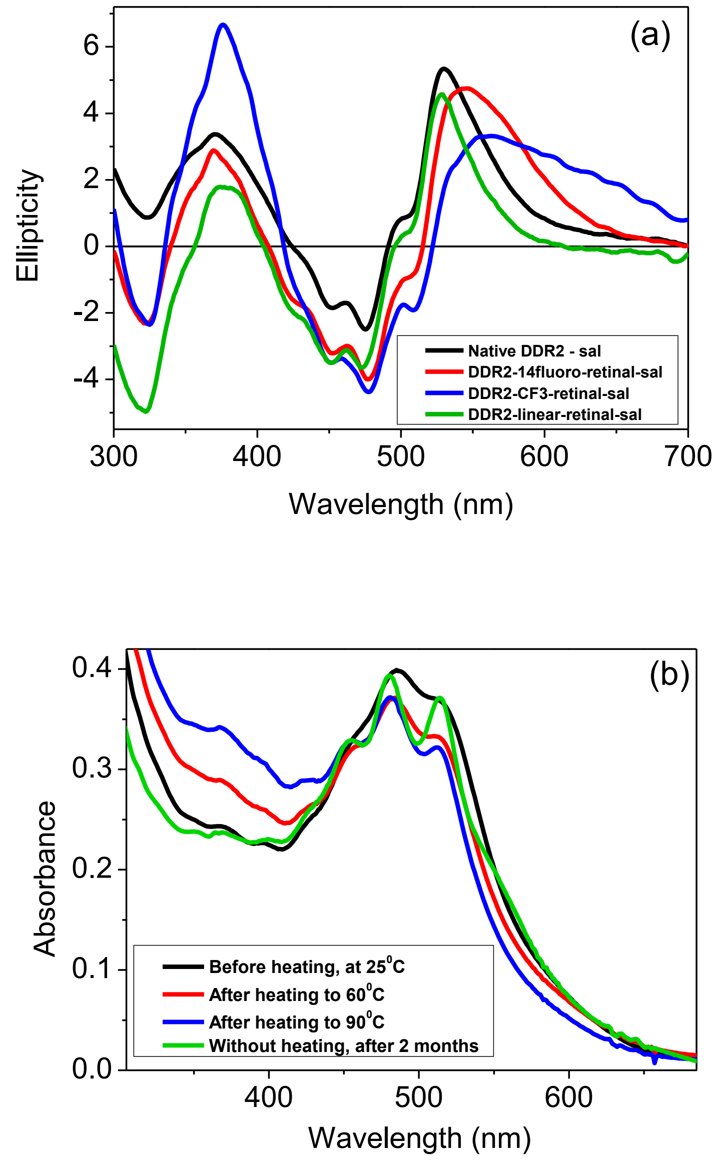

Figure S11: (a) The CD spectra of complexes formed between artificial DDR2 pigments shown in Figure 5 and Sal. The same for native DDR2-Sal complex is shown for comparison. (b) Absorption spectra of DDR2-Sal complex at 25 °C, at 60 °C and at 90 °C. Those recorded after keeping the sample at 25 °C for two months are also shown.

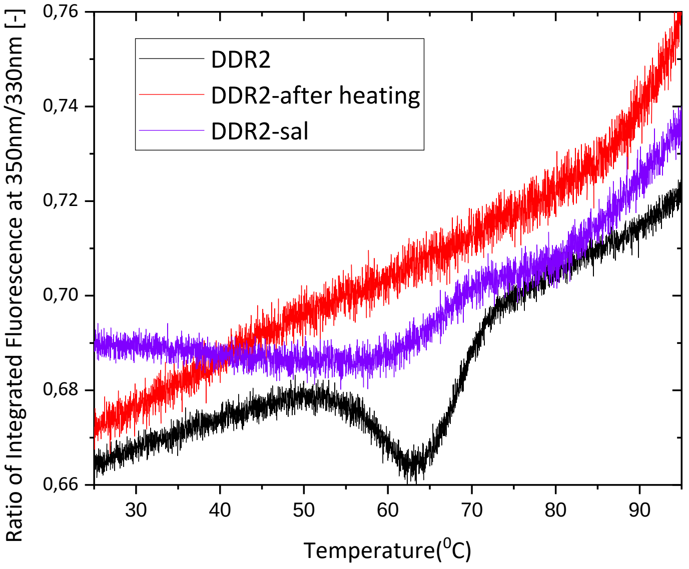

Figure S12: Differential scanning fluorimetry (DSF) of DDR2, DD2-Sal and the heat-treated DDR2 (heated to 80 °C for 10 minutes, followed by cooling to 25 °C). The ratios of emission intensity at 350 nm to that of 330 nm of tryptophan (Trp) residue are plotted against increase in temperature.
